# Design and Performance Evaluation of Integrating the Waste Heat Recovery System (WHRS) for a Silicon Arc Furnace with Plasma Gasification for Medical Waste

**DOI:** 10.3390/e25040595

**Published:** 2023-03-31

**Authors:** Yuehong Dong, Lai Wei, Sheng Wang, Peiyuan Pan, Heng Chen

**Affiliations:** 1 State Key Laboratory of Clean and Efficient Coal-Fired Power Generation and Pollution Control, China Energy Science and Technology Research Institute Co., Ltd., Nanjing 210023, China; 2 Beijing Key Laboratory of Emission Surveillance and Control for Thermal Power Generation, North China Electric Power University, Beijing 102206, China; l.wei@ncepu.edu.cn (L.W.); heng@ncepu.edu.cn (H.C.)

**Keywords:** waste heat recovery, plasma gasification, silicon arc furnace, medical waste, system integration

## Abstract

A hybrid scheme integrating the current waste heat recovery system (WHRS) for a silicon arc furnace with plasma gasification for medical waste is proposed. Combustible syngas converted from medical waste is used to drive the gas turbine for power generation, and waste heat is recovered from the raw syngas and exhaust gas from the gas turbine for auxiliary heating of steam and feed water in the WHRS. Meanwhile, the plasma gasifier can also achieve a harmless disposal of the hazardous fine silica particles generated in polysilicon production. The performance of the proposed design is investigated by energy, exergy, and economic analysis. The results indicate that after the integration, medical waste gave rise to 4.17 MW net power at an efficiency of up to 33.99%. Meanwhile, 4320 t of the silica powder can be disposed conveniently by the plasma gasifier every year, as well as 23,040 t of medical waste. The proposed design of upgrading the current WHRS to the hybrid system requires an initial investment of 18,843.65 K$ and has a short dynamic payback period of 3.94 years. Therefore, the hybrid scheme is feasible and promising for commercial application.

## 1. Introduction

Solar energy is a widely distributed renewable energy and becoming increasingly popular for power generation. Photovoltaics (PV) is at present the most used and cost-effective technology of solar energy, which converts sunlight into electricity, directly based on the photovoltaic effect. In the last decade, PV production witnessed great growth. In 2021, solar PV capacity increased by 17% globally, accounting for ~60% of the total renewable power expansion [1]. In order to meet the fast-growing solar PV demand, global production of PV-related products is expected to more than double by 2030 [1]. Today, China dominates the global solar PV supply chains and contributes to an 80% decline in the price of solar panels, making solar PV an affordable electricity generation technology [2,3].

Crystalline silicon modules have dominated the current solar PV market at more than 95% of the installed capacity in the last five years [4]. Solar PV manufacturing is energy-intensive and mostly powered by fossil fuels. Polysilicon production is the largest energy-consuming segment of the solar PV supply chain, accounting for up to ~40% of the total energy consumption [4]. The first stage of polysilicon production is to extract metallurgical-grade silicon by melting quartz ore and reducing silica in a large electric arc furnace, which requires a great deal of heat at a high temperature (~2000 °C) and a lengthy time [5,6]. Metallurgical-grade silicon of 98% silicon purity is the fundamental material of subsequent silicon products [2,7]. Generally, 10 to 13 MWh of electricity is needed to produce one ton of metallurgical-grade silicon, but only ~30% of the total energy input is contained in the silicon product, whereas the rest of the energy is taken away as thermal energy by the off gas and the cooling water [6,8]. The temperature of the exhaust gas leaving the arc furnace mainly depends on the furnace load and air excess, at 500 to 700 °C. Therefore, there is great potential to utilize the waste heat in the exhaust gas, and a heat recovery steam generator (HRSG) with a steam turbine is a suitable and cost-effective method [9]. Currently, many large-scale silicon arc furnaces are equipped with some sort of waste heat recovery system (WHRS), and the generated electricity can offset some of their power consumption. Metallurgical-grade silicon is subsequently purified into solar-grade polysilicon of 6–13 N purity [7,10].

Solar PV systems have obvious superiority to traditional power generation methods due to near-zero emission of atmospheric pollutants and greenhouse gases during operation, but the vast majority of their environmental burdens caused by solar PV are released during their manufacturing processes [11,12]. For instance, a lot of silica particles are formed in the silicon arc furnace and entrained by the exhaust gas, which needs to be gathered and removed before going into the atmosphere [13,14]. These particles are quite small in size, and their high specific surface area and volume result in easy contamination and difficult transportation. Emission or leakage of these fine particles aggravates atmospheric pollution and is hazardous to human health.

Due to good stability, silica cannot be easily tackled by common chemical methods, although melting followed by consolidation seems to be an effective way. Nowadays, plasma gasification is regarded as a superior and promising waste to energy (WTE) technique of solid waste [15,16,17,18].

Direct current (DC) plasma is widely considered due to its better stability and load adaptability than other types of plasma. In a DC plasma gasifier, strong DC electric arc is created by electrodes, resulting in thermal plasma and a high-temperature environment, which can destroy nearly all chemical bonds in the substances, releasing free electrons, ions, radicals, and molecules, allowing many reactions that cannot proceed in normal conditions to occur [19,20]. Consequently, the input organics are quickly decomposed into their component elements, which subsequently react to form a synthetic gas mostly consisting of H_2_, CO, CH_4_, and some other light hydrocarbons, and meanwhile, the inorganics are completely melted and transformed into inert and nontoxic glassy slags [21,22,23]. Plasma gasification has two remarkable advantages: (1) producing combustible syngas of high calorific value, as a clean and valuable fuel for power generation; (2) disposing of a broad variety of solid wastes safely and harmlessly, especially some hazardous wastes, while consolidating solid residues [18,24,25].

Plasma gasification has been widely considered in the treatment of municipal solid wastes (MSW) as an alternative to traditional incineration and landfill, due to its outstanding environmental benefits and flexibility [16,18,26,27,28]. The disposal fee of wastes and electricity selling are the major sources of income for the power plant based on plasma gasification. However, at present, plasma gasification plants are quite scarce, owing to their high investments and operating costs, and therefore, energy conversion efficiency and economic viability are emphasized.

As far as net electrical efficiency is concerned, a plasma gasification power plant containing only one single-stage steam turbine or gas turbine does not obviously perform better than conventional incineration plants. Using MSW as the feedstock, the net electrical efficiency of a plasma gasification power plant based on an individual steam cycle (Rankine cycle) is only about 14 to 21%, and utilization of a single gas turbine has the efficiency of 13 to 24%, compared to 20 to 30% of direct incineration [24,27,29,30,31,32,33]. Therefore, an integrated plasma gasification combined cycle (IPGCC) is essential for efficiency improvement. In an IPGCC plant, the direct conversion of chemical energy of syngas into electricity is conducted by a gas turbine, and meanwhile, a steam cycle is established for heat recovery from the exhaust gas of the gas turbine, thereby promoting overall power generation and economic competitiveness. The net electrical efficiency of an IPGCC plant can be close to or even exceed 30% [34,35]. Minutillo et al. [24] developed a thermochemical model of an IPGCC plant and pointed out that the system efficiency of power generation could be up to 31%. Montiel-Bohórquez et al. [20] assessed the technical and economic performance of an IPGCC plant fueled with MSW, finding out its highest efficiency was 32.49%, and one-third of the total gross power output was from the steam turbine.

Recently, the development of healthcare facilities and the breaking COVID-19 pandemic made medical waste management a great environmental issue [36,37,38]. Medical waste is mainly composed of plastics, paper, textiles, glass, and some organics, this means that they have similar characteristics to typical MSW [39,40,41]. But due to the relatively low moisture content, high volatile content, and high lower calorific value (LHV) compared to MSW, medical waste is more worth exploiting in view of WTE [37,38]. Furthermore, because medical waste contains some infectious, pathological, chemical, pharmaceutical, or cytotoxic matters, there are potential risks of environmental pollution and infection, which lead to high disposal costs [30,42,43].

In this study, medical waste rather than common MSW is taken as the major feedstock in the plasma gasifier [36,38]. The plasma gasifier is designed to be built close to the silicon arc furnace so that simultaneous disposal of medical waste and silica powder can be performed.

Over the past several decades, a WHRS for power generation based on the steam cycle has been extensively investigated and applied in many industrial processes. The concept of incorporating a conventional WHRS into the gasification system also has been proposed, because one of the HRSGs could be saved by sharing the equipment, thereby lowering the overall costs and land occupation. Chen et al. [44] designed a novel medical WTE system based on plasma gasification integrated with an MSW incineration plant, using exhaust gas from the gas turbine to heat the live steam and feed water in the incineration plant, thereby increasing WTE efficiency up to 37.83%. Yang et al. [45] proposed and techno-economically assessed a WTE process based on combined heat and power plant and intermediate pyrolysis technology, finding that the levelized cost of electricity was £0.063/kWh. In the authors’ previous work [46], plasma waste gasification was integrated with a coal-fired power plant, promoting WTE efficiency by feeding the syngas directly into the coal-fired boiler. However, in the previous literature, the gasification system is usually integrated with large-scale power plants, so that the thermal energy contained by the syngas can be utilized at a high temperature, benefiting its efficiency. A WHRS for small-scale industrial boilers generating live steam of relatively low parameters is seldom considered. On the other hand, there is hardly any research on the treatment of the silica powder generated in polysilicon production by plasma gasification, because the melting of SiO_2_ brings down the gasification efficiency. Therefore, the economic performance of integrating the WHRS of a silicon arc furnace with plasma gasification is still questionable.

In this work, in view of the expanding polysilicon production and increasing demand for medical waste treatment, a design that integrates the current WHRS of a silicon arc furnace with plasma gasification for medical waste is proposed. The advantage of this integration mainly includes: (1) The heat recovered in the plasma gasification system is exploited for extra heating of the steam and feed water in WHRS, thereby promoting WTE efficiency of the medical waste without affecting the power output by the exhaust gas from the silicon arc furnace. (2) The silica powder collected from the flue gas leaving the silicon furnace can be fed into the plasma gasifier and consolidated into vitrified slags, thereby avoiding pollution caused by the leakage of fine particles. (3) An HRSG system in a typical IPGCC scheme can be substituted by the existing equipment in a WHRS, so that investment, operational costs, and land occupation of the plasma gasification system can be significantly lowered.

## 2. System Description

A 33 MVA submerged arc furnace producing metallurgical-grade silicon with its current WHRS was selected as the reference plant, which is now operational at a large-scale manufacturing base of silicon PV in northwestern China. The manufacturing base has 32 silicon arc furnaces and dozens of polysilicon purification and monocrystalline silicon production lines. At present, each arc furnace has already been equipped with WHRS for power generation. The furnaces and WHRS have both been operating reliably for the past five years, and the operating parameters collected online coincided well with design values. In this study, the design diagrams and data provided by the manufacturers were used for analysis. The existing WHRS and the proposed WHRS integrated with plasma gasification for medical waste are respectively described in Section 2.1 and Section 2.2.

### 2.1. Current WHRS for the Silicon Arc Furnace

**Figure 1:**
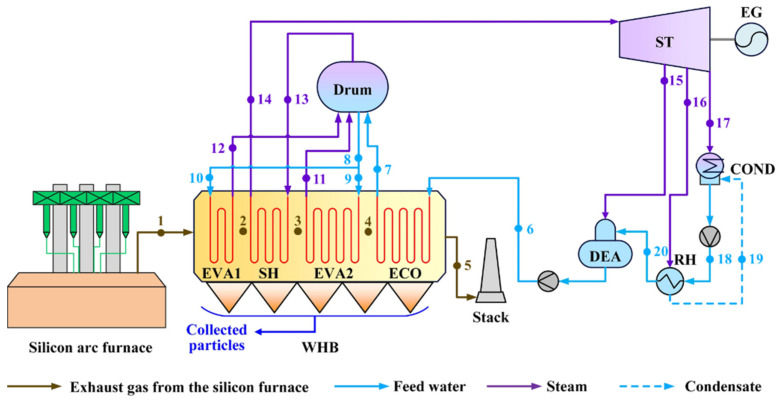
Diagram of the current WHRS for the silicon arc furnace.

Figure 1 shows the current WHRS for the silicon arc furnace. Quartz ore that mainly consists of SiO_2_ is intermittently fed into the submerged arc furnace and reduced by carbon reductants (coke) at ~2000 °C to produce polysilicon. The major chemical reactions are as follows [6]:

2C(s) + SiO_2_(s) → Si(l) + SiO(g) + CO(g), (1)

SiO(g) + 2C(s) → SiC(s) + CO(g),(2)

SiC(s) + SiO_2_(s) → Si(l) + SiO(g) + CO(g), (3)

These reactions occur in the packed bed and the required heat is provided by the arc created by three AC electrodes that are half submerged in the raw material. Molten silicon is taken out through the tapping at the bottom, while the hot exhaust gas continuously flows upward and leaves the furnace through the top hole. Table 1 lists the main gaseous components of the exhaust gas. The average temperature of the exhaust gas is ~650 °C, making its heat worth recovering. Meanwhile, the exhaust gas has 4 to 10 g/m^3^ of fly ash, and the main components of the fly ash are listed in Table 2. Fine amorphous silica particles dominate in the fly ash, and have an average particle size of less than 1 μm.

As depicted in Figure 1, current WHRS is composed of a waste heat boiler (WHB) for waste heat recovery and steam generation, a steam turbine (ST) driven by the live steam, an electricity generator (EG) for power generation, and heat regeneration equipment. Their basic parameters are referred by the values in Table A1 and Table A2 in Appendix A. The exhaust gas leaving the silicon arc furnace flows through WHB and is cooled from 650 °C to 187.3 °C, and meanwhile, the feed water from the outlet of the deaerator (DEA) at 104.8 °C/4.02 MPa is heated to a superheated steam at 450 °C/3.82 MPa. Due to the high content and small particle size of the fly ash in the gas flow, efficient and uninterrupted ash removal from heat transfer surfaces is necessary in WHB. Mechanical striking using steel balls is applied and the falling ash is collected by the ash hoppers at the bottom.

The feed water delivered to WHB is first heated in the economizer (ECO) to approximate saturated water. Water/steam separation is performed in the drum. Some of the saturated water from the drum flows into the evaporator 2 (EVA2) between the superheater (SH) and ECO, and the rest of the saturated water is sent to EVA1 at the inlet of WHB. The purpose of EVA1 is to cool the high-temperature gas rapidly in view of slagging prevention. The saturated steam separated by the drum is sent into SH for final heating and then flows into ST.

### 2.2. Proposed WHRS Integrated with Plasma Gasification

The current WHRS can be integrated with a plasma gasification system fueled with medical waste, exploiting the heat recovered from syngas treating processes to further raise its power output. The combustible syngas generated by gasification is used for power generation through the gas turbine (GT). In the meantime, the collected silica powder is fed into the gasifier together with medical waste and ends up in vitrified slags that are harmless and transportable.

**Figure 2:**
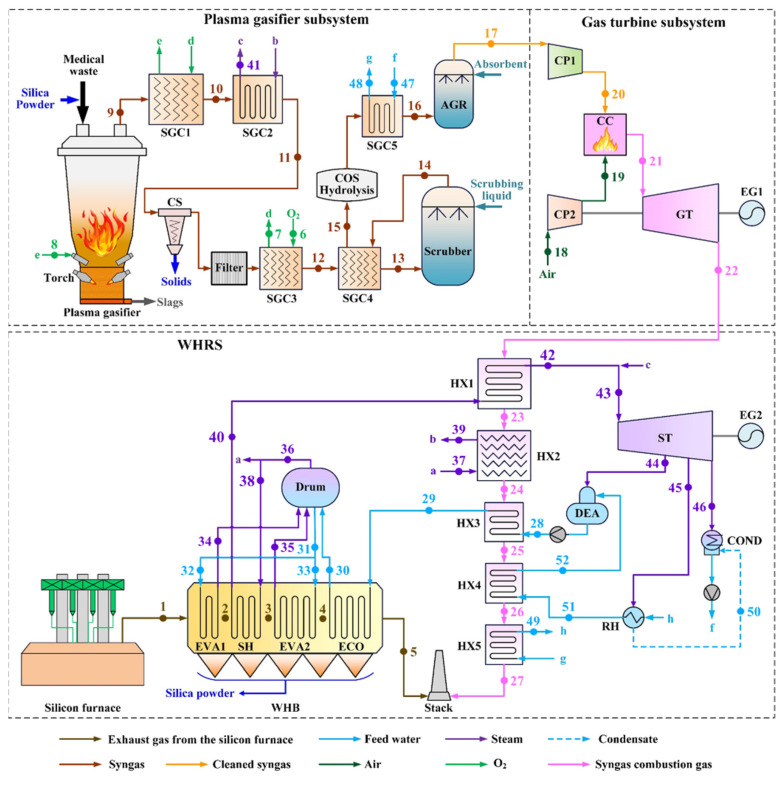
Diagram of the proposed WHRS integrated with plasma gasification for medical waste.

This proposed hybrid scheme is illustrated in Figure 2. From the perspective of power generation based on plasma gasification, a combination with WHRS is an alternative to conventional IPGCC, making full use of the syngas and combustion gas and requiring lower capital investment.

The plasma gasification system can be roughly divided into two parts, the plasma gasifier subsystem, and the gas turbine subsystem.

The plasma gasifier subsystem includes the DC plasma gasifier and syngas conditioning equipment [20]. The medical waste and the collected silica powders are fed from the top of the gasifier, and the organic components quickly decompose when being heated, generating volatiles. O_2_ separated from the air is injected into the bottom of the gasifier as the oxidizing agent of the gasification process, and the extremely high temperature environment (~4000 °C) created by plasma torches transforms the residual carbon, hydrogen, and other combustible elements into micro-molecular gases. The remaining inorganic solids, including the fine silica particles in the feedstock, are completely melted, and the effluent slags are cooled and solidified.

The syngas formed in the gasifier contains various high calorific value components, making it a good fuel. However, the formation of contaminants, such as particulates, condensable hydrocarbons, sulfur compounds, nitrogen compounds, halides, and trace heavy metals, is inevitable in gasification, and thus the raw syngas needs to be cleaned to meet stringent emission regulations and protect the downstream equipment from fouling, corrosion, and erosion [47,48]. There is a multitude of technologies for syngas purification. Conventional syngas cleaning equipment includes the cyclone separator (CS), wet scrubber, carbonyl sulfide (COS) hydrolysis, acid gas removal (AGR), and filters [47,49]. Syngas needs to be properly cooled and heated in order to meet the temperature requirements of different cleaning processes [48].

Heat recovery in syngas conditioning is essential for attaining system efficiency, and thus heat exchangers are required. As shown in Figure 2, the raw syngas is first cooled in a gas-gas heat exchanger, named syngas cooler 1 (SGC1). O_2_ entering the plasma gasifier needs to be well-preheated to facilitate gasification. In this scheme, O_2_ has two-stage preheating, and SGC1 is used for the second stage. SGC2 heats a steam flow from WHRS and supplies superheated steam to ST directly. Syngas from SGC2 flows through CS and a filter to remove bulk particles. CS is a widely used inertial separation device and is able to operate in a wide temperature range while requiring low energy [48,50]. In view of syngas, a temperature of 300 to 500 °C benefits the operation of CS and filters because particulate matters can stay in the solid state, avoiding problems caused by melting or moisture absorption.

The filtered syngas is then used for the first-stage preheating of O_2_ in SGC3. SGC4 is placed downstream of SGC3, cooling the syngas further to ~130 °C, while reheating the syngas at the outlet of the wet scrubber to 250.0 °C. Water scrubbing is an easily-operated and effective method for syngas decontamination, removing NH_3_ compounds, halides, fine particles, some H_2_S, and other trace contaminants simultaneously [47,49]. However, a large flow of scrubbing water is required so that the syngas is rapidly cooled to its dew point temperature, ensuring the finest particles can be removed by acting as the nuclei for condensation [51].

Sulfur compounds are the main residual contaminants in the scrubbed syngas. Although most sulfur in the feedstock is converted to H_2_S in gasification, about 3 to 10% of the sulfur is converted to carbonyl sulfide, which is the main organic sulfur component and cannot be efficiently removed by the downstream AGR due to its low solubility in most solvents. Therefore, a catalytic hydrolysis reactor is set up to convert over 99% of COS in the syngas to H_2_S, according to the flowing reaction [47,52].

COS(g) + H_2_O(g) ↔ H_2_S(g) + CO_2_(g), (4)

The scrubbed syngas needs to be reheated to ~250 °C in view of the efficiency of alumina-based catalysts used in COS hydrolysis [53,54]. The conversion of COS to H_2_S is an exothermic reaction, technically, but the passing of the syngas through COS hydrolysis can be regarded as an isothermal process in heat calculation due to the low concentration of COS.

AGR is the endmost syngas cleaning process, using regenerative solvents in an absorber column to remove various sulfur-bearing gases, including H_2_S and SO_2_ surviving from the wet scrubbing, H_2_S converted from COS by hydrolysis, and some organic sulfur compounds [55,56]. Most of the common chemical solvents, such as piperazine-activated methyl diethanolamine (MDEA) and aqueous alkaline salt solutions, are effective over a wide range of acid gas concentrations at near-room temperatures, and thus AGR is typically designed to operate at slightly above the ambient temperature [56]. In order to minimize the heat loss due to AGR, the syngas from COS hydrolysis is used to heat the low-temperature condensate from condenser (COND) of WHRS in SGC5.

In the gas turbine subsystem, the cleaned syngas is sent to compressor 1 (CP1) for compression, and meanwhile, the feeding air is compressed in CP2. Syngas is burned in the combustion chamber (CC), and the formed high-temperature and -pressure combustion gas enters GT, generating power by electric generator 1 (EG1).

The exhaust gas of GT still has a high temperature and is delivered to the WHRS of the silicon arc furnace for heat exploitation, so as to save HRSG equipment in the conventional IPGCC scheme. The combustion gas leaving GT flows through gas coolers HX1 to HX5 successively.

The live steam supplied by WHB is further heated in HX1 before entering ST. HX2 is to heat a saturated steam flow from the drum, which is subsequently sent to SGC2 for final heating. HX4 and HX5 are used for additional heating of the feed water in the heat regeneration subsystem so as to save the extracted steam from ST. The condensate from COND flows through SGC5, HX5, RH, and HX4 successively before entering DEA. At last, the temperature of the feed water into WHB is promoted by HX3.

Via integration with plasma gasification, the steam cycle in WHRS is largely assisted by the heat recovered from the syngas and combustion gas, and thus its power generation capacity is improved. Meanwhile, the combination with WHRS makes it possible for the plasma gasification power generation system to save some high-cost components compared to conventional IPGCC systems. Furthermore, with a view to waste management, the troublesome silica fines generated by polysilicon production can be handled by the plasma gasifier harmlessly.

## 3. Methodology

The models and analysis methods used in this work are described in this chapter. The current WHRS, a plasma gasifier, and a gas turbine system are individually simulated, and the obtained parameters are compared with the operational data or data from references to validate the reliability of these models.

### 3.1. Analysis Methods

#### 3.1.1. Energy Analysis

The power generation efficiency (*η*_p,wh_, %) of WHRS is defined as follows.

(5) $$\eta_{p,wh}=\frac{P_{net,wh}}{Q_{rec,wh}},$$

where *P*_net,wh_ is the net power generation by the exhaust gas from the silicon arc furnace, MW. *Q*_rec,wh_ is the thermal energy recovered from the exhaust gas, MW. They are respectively calculated as follows.

(6) $$P_{net,wh}=P_{gr,wh}-P_{ax,wh},$$

(7) $$Q_{rec,wh}=m_{eg}\times \left(h_{fg,in}-h_{fg,out}\right),$$

where *P*_gr,wh_ is the gross power output by ST, MW. *P*_ax,wh_ is the estimated power consumption by the auxiliaries in WHRS, MW. *m*_eg_ is the flow rate of the exhaust gas from the silicon arc furnace, kg/s. *h*_fg,in_ and *h*_fg,out_ are specific enthalpies of the inlet and outlet flue gas of WHB, respectively, kJ/kg.

In the plasma gasifier, solid waste is used as the feedstock, and air and steam are used to assist gasification. Plasma torches are the main heat source and power consumer in the gasifier, and the torch thermal efficiency is estimated to be 86%. The plasma gasification efficiency (*η*_pg_, %) is calculated as follows [57].

(8) $$\eta_{pg}=\frac{m_{syn}\times {LHV}_{syn}}{m_{waste}\times {LHV}_{waste}+\frac{P_{tor}}{\eta_{tor}\times \eta_{e}}},$$

where *m*_syn_ is the flow rate of the syngas generated by plasma gasification, kg/s. LHV_syn_ is the lower heating value of the syngas, MJ/kg. *m*_waste_ is the feed rate of waste into the plasma gasifier, kg/s. LHV_waste_ is the lower heating value of the waste, MJ/kg. *P*_tor_ is the power consumption by the plasma torches, MW. *η*_tor_ is torch thermal efficiency, %. *η*_e_ is the overall power generation efficiency of the plasma gasification plant, %, considered as 35% according to the average level of IPGCC plants [57].

In the hybrid system, assuming the power generation by the exhaust gas of the arc furnace (*P*_net,wh_, MW) is constant, the net power generation by the plasma gasification of medical waste (*P*_net,pg_, MW) and its efficiency (*η*_p,pg_, %) are calculated as follows.

(9) $$P_{net,pg}=P_{net,tot}-P_{net,wh},$$

(10) $$\eta_{p,pg}=\frac{P_{net,pg}}{m_{mw}\times {LHV}_{mw}},$$

where *P*_net,tot_ is the total net output by ST and GT, MW.

The overall power generation efficiency of the hybrid system (*η*_p,tot_, %) is calculated as follows.

(11) $$\eta_{p,tot}=\frac{P_{net,tot}}{Q_{rec,wh}+m_{mw}\times {LHV}_{mw}},$$

Power consumption by auxiliaries in WHRS is estimated to be 15% of the current gross power output by ST. Power consumption by the syngas conditioning equipment is estimated to be 5% of the gross power output of GT. In the plasma gasifier, power consumption by O_2_ separation from the air is estimated to be 0.261 kWh/kg_pure O2_ [24]. Torch thermal efficiency when O_2_ is used for gasification assistance is estimated to be 90%.

#### 3.1.2. Exergy Analysis

Exergy is an indicator of both the quantity and quality of the energy, which can be used to assess the utilization potential of an energy source and the performance of a system [58,59]. The exergy input by the exhaust gas of the silicon arc furnace (*EX*_fg_, MW) and by the medical waste (*EX*_mw_, MW) are respectively calculated as follows [44].

(12) $${EX}_{fg}=m_{fg}\times \left[h_{fg,in}-h_{fg,0}-T_{0}\times \left(s_{fg,in}-s_{fg,0}\right)\right],$$

(13) $${EX}_{mw}=m_{mw}\times {LHV}_{mw}\times \left(1.0064+0.1519\times \frac{\omega_{H}}{\omega_{C}}+0.0616\times \frac{\omega_{O}}{\omega_{C}}+0.0429\times \frac{\omega_{N}}{\omega_{C}}\right),$$

where *T*_0_ is the environmental temperature, assigned as 293.15 K. *h*_fg,0_ is specific enthalpy of the flue gas at *T*_0_, kJ/kg. *s*_fg,in_ and *s*_fg,0_ are specific entropies of the flue gas at the inlet state and at *T*_0_, respectively, kJ/(kg·K). *ω*_C_, *ω*_H_, *ω*_O_, and *ω*_N_ are the mass contents of elements C, H, O, and N in the medical waste.

There is an exergy balance in energy processes, which can be applied to the components, subsystem, and entire system.

(14) $${EX}_{in}+W_{in}={EX}_{out}+W_{out}+{EX}_{des},$$

where *EX*_in_ and *EX*_out_ are the exergy input and output, MW. *W*_in_ and *W*_out_ are the work input and output, MW. *EX*_des_ is the exergy destruction, MW.

The exergy efficiency of power generation by plasma gasification of the medical waste (*η*_ex,pg_, %) and the overall exergy efficiency of the hybrid system (*η*_ex,tot_, %) are calculated as follows.

(15) $$\eta_{ex,pg}=\frac{P_{net,pg}}{{EX}_{mw}},$$

(16) $$\eta_{ex,tot}=\frac{P_{net,tot}}{{EX}_{fg}+{EX}_{mw}},$$

#### 3.1.3. Economic Analysis

The main economic income of the integrated system includes disposal fees for medical waste, electricity selling, and slag selling. Economic analysis is conducted based on the assumptions given in Table 3. The construction period includes 0.5 years for the reconstruction of the current WHRS, assuming in the second year, which causes a decrease in electricity generation of that year, and thus compensation for the income decrease is considered.

Upgrade of the current WHRS to the proposed hybrid system requires new equipment, and the cost of these components is estimated based on the methods in Table 4 and Table 5. The scaling-up methods in Table 5 are conducted as follows [61].

(17) $$C={C_{0}\times (\frac{S}{S_{0}})}^{ƒ}$$

where *C*_0_ is the basic cost of the reference equipment, k$; *C* is the capital cost of the target equipment, k$; *S*_0_ is the basic scale of the reference equipment; *S* is the scale of the target equipment; ƒ is the scale factor.

The dynamic payback period (DPP) of the integration projected is an important and widely used economic performance indicator, representing the least necessary time to recover the initial investment. Meanwhile, the net present value (NPV, k$), referring to the variation of the cash inflow over the lifetime of the project, is usually employed in DPP calculation, in order to consider the discounting risk of cash. NPV and DPP are estimated as follows [44,66].

(18) $$NPV=\sum_{y=1}^{k}\frac{C_{inflow}-C_{outflow}}{{\left(1+r_{dis}\right)}^{y}},$$

(19) $$\sum_{y=1}^{DPP}\frac{C_{inflow}-C_{outflow}}{{\left(1+r_{dis}\right)}^{y}}=0,$$

where *k* is the lifetime of this project, assigned as 25 years. *y* is the year number in the lifetime. *r*_dis_ is the discount rate, estimated as 12%. *C*_inflow_ and *C*_outflow_ are the cash inflow and cash outflow in year *y*, k$.

A shorter DPP and a higher NPV are favored for a project.

### 3.2. Model Development and Validation

**Figure 3:**
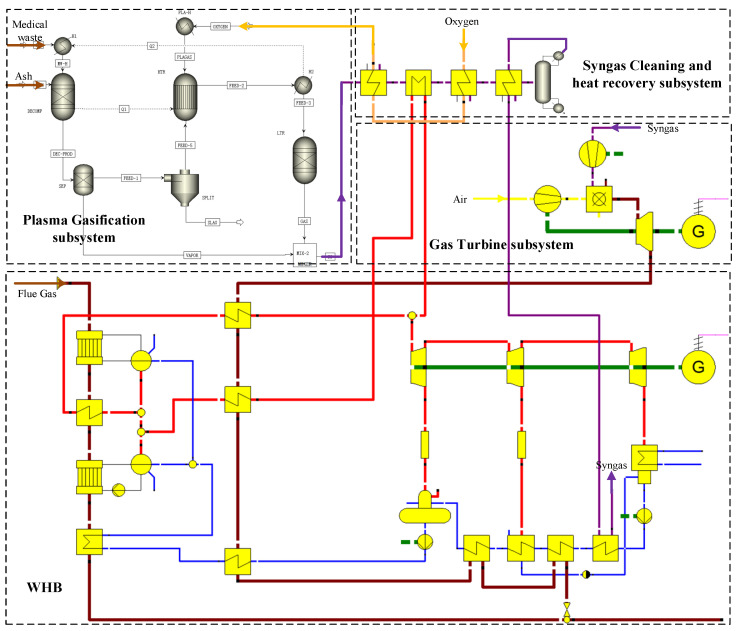
Models for simulation of the proposed WHRS integrated with plasma gasification based on EBSILON Professional and Aspen Plus.

WHRS and the plasma gasification system are mainly modeled and simulated using the EBSILON Professional platform. The behavior of each modeled equipment is described by thermodynamic laws, and the thermodynamic cycle is tackled through a group of linear equations, which are solved iteratively. Accordingly, parameters of the system with low uncertainties can be derived. Moreover, the plasma gasification process is modeled by Aspen Plus, due to its specialty in chemical simulation. Figure 3 illustrates the established models of the proposed hybrid system.

The composition of medical waste varies. One type of medical waste is selected for modeling and its properties are shown in Table 6.

The current WHRS for the 33 MVA submerged arc furnace illustrated in Figure 1 is modeled to validate the reliability of models, and the boundary conditions are from its design parameters. Simulation results obtained by iteration are compared with the design or operating parameters in Table A1 in Appendix A. Detailed parameters of stream flows in Figure 1 are listed in Table A2.

In order to validate the reliability of models for the plasma gasifier and equipment in GT section, a plasma gasifier fueled with solid waste is simulated by Aspen Plus, and a GT system containing CP, CC, and GT is simulated by EBSILON Professional. The simulation results are listed in Table A3 and Table A4, compared with the reference values [68,69]. Waste power output by GT has excluded the power consumed by CP to compress air because they are coaxial.

The comparison reveals good reliability of the established models so that precise parameters of the proposed system can also be obtained based on these models.

## 4. Results and Discussion

### 4.1. Parameters of the Proposed Hybrid System

#### 4.1.1. Plasma Gasifier Subsystem

Table 7 presents the main parameters of the plasma gasifier in the hybrid system. The silica powder is fed at 0.15 kg/s, so that most of the silica fines collected in WHB can be treated harmlessly. However, power consumption by the torches increases because extra energy is required to melt SiO_2_. The raw syngas leaving the gasifier has an initial temperature of 818.4 °C and a lower heating value of 10.57 MJ/kg.

Along the conditioning processes, the syngas has significant temperature changes only in SGCs, wet scrubber, and AGR, and other procedures can be regarded as nearly isothermal. Composition change of the syngas is inconsiderable and neglectable from the perspective of energy calculation, due to relatively low concentrations of the pollutants. The outlet temperature of the wet scrubber is 47.1 °C, approximately equal to the syngas dewpoint. The outlet temperature of AGR is treated as 40 °C, a little higher than typical environmental temperature, which means the syngas witnesses moisture condensation, and the condensates are left in AGR. The main parameters of SGCs, wet scrubber, and AGR obtained by simulation are listed in Table 8.

#### 4.1.2. Gas Turbine Subsystem

Table 9 presents the main parameters of the gas turbine subsystem. Before combustion, the clean syngas is compressed in CP1, and simultaneously, O_2_ is fed at a ratio of 9.50 kg/s and compressed in CP2. The compressed gases of 1.42 MPa are mixed and burned in CC to form hot combustion gas at 1303.3 °C, which subsequently drives GT for power generation and has a net power output of 4.70 MW. The exhaust gas leaving GT is still at a high temperature of 666.6 °C.

#### 4.1.3. WHRS for the Silicon Arc Furnace

In the proposed WHRS integrated with plasma gasification, extra heat provided by the exhaust gas of GT and the syngas is used to assist in the temperature promotion of the steam entering ST and the water entering WHB. Table 10 lists the main parameters of the new WHRS, and Table 11 lists the parameters of the heat exchangers in WHB.

As shown in Figure 2, in the hybrid scheme, the saturated steam leaving the drum is divided into two flows. One steam flow of 3.30 kg/s is heated in HX2 and SGC2 successively, from 249.2 °C to 480.0 °C. The other steam flow of 6.65 kg/s is sent to SH in WHB as before, but subsequently further heated in HX1 to 480.0 °C. The two superheated steam flows merge before entering ST. Compared with the current design, the power generation capacity of ST in the integrated WHRS is greatly promoted by the increase in temperature and flow rate of the inlet steam, from 8.08 MW to 10.21 MW. The steam temperature in WHB is controlled no higher than 450 °C, in view of the anti-fouling requirements of the heat transfer surfaces.

Table 12 and Table 13 list the parameters of HX1 to 5 and the heat regeneration equipment. Because of auxiliary heating by HX3 to 5, the amount of steam extracted from ST and fed into RH and DEA is reduced from 1.00 kg/s to 0.05 kg/s, benefiting power generation by ST, and the temperature of the feed water entering WHB increases from 104.8 °C to 167.0 °C. Detailed parameters of the stream flows in the proposed system are listed in Table A5.

### 4.2. Energy Performance

The energy performance of the integrated system and current WHRS is examined and compared in Table 14. After the integration, the heat recovered from the exhaust gas leaving the silicon arc furnace is invariable. Medical waste is fed into the plasma gasifier and supplies extra energy by its conversion into combustible syngas. From this, 4.70 MW of power could be generated by GT, and meanwhile, ST has an increase of 2.13 MW in its gross power output. After the integration, auxiliary power consumption increases from 1.21 MW to 3.82 MW, due to the plasma torches, O_2_ separation, syngas conditioning equipment, and CP1. Plasma torches are the biggest power consumer in the proposed WHRS because a great deal of heat is required to maintain a high-temperature environment in the gasifier, especially when high-melting SiO_2_ exists, which is inevitable when consolidating silica fines using thermal methods. To sum up, 4.17 MW power can be attributed to plasma gasification for medical waste in this case, and the net power generation efficiency of medical waste exploitation is up to 33.99%, close to the power generation efficiency of conventional IPGCC plants, despite the extra heat caused by SiO_2_ melting in gasification. Overall power generation efficiency of WHRS increases from 28.19% to 30.13%.

**Figure 4:**
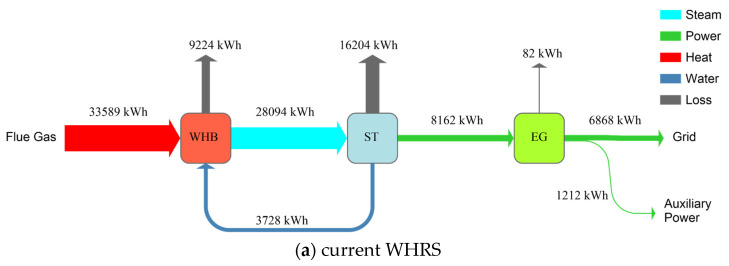

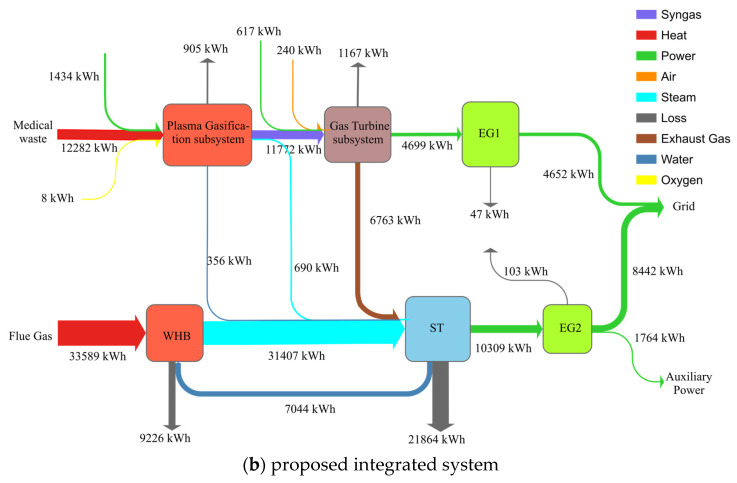
Energy flows in the current WHRS and the proposed integrated system.

Figure 4 illustrates detailed energy flows in the current WHRS and the proposed integrated system. The exhaust gas from WHB and exhaust steam from ST are major causes of energy loss both in the current WHRS and the proposed system. In the proposed system, 1.05 MW and 6.76 MW of waste heat are recovered from the syngas conditioning processes and the exhaust gas from GT, respectively, to assist power generation by ST. The power generation efficiency through the steam cycle can also be promoted because the steam temperature entering ST increases from 450 °C to 480 °C.

### 4.3. Exergy Performance

Table 15 lists the exergy analysis results of the proposed integrated system and the current WHRS. Exergy input by the exhaust gas from the arc furnace is unaltered and regarded as 100%, and in the hybrid scheme, gasification of medical waste offers an increase of 87.75% in exergy input. Table 16 displays detailed exergy destruction in the components of the system.

Exergy destruction in the current system mainly occurred in WHB and ST. In the proposed system, exergy destruction in WHB slightly decreases because of the higher average temperature for heat absorption. The inevitable increase in exergy destruction in ST and EG2 is caused by the promotion of power output. Among the newly added heat exchangers, HX2-5 working at low temperatures have relatively high exergy destruction. Besides, the gasifier and CC also lead to great exergy destruction. Overall, after integration, the total exergy destruction more than doubled, mainly due to the addition of necessary equipment for plasma gasification. The gasification and combustion reactions are responsible for the relatively low exergy efficiency of power generation by medical waste. So that the overall exergy efficiency of the integrated system is lower than the current system.

### 4.4. Economic Performance

Economic benefits are important advantages of the proposed integrated WHRS. When the plasma gasification system operates alone, in order to exploit heat from the exhaust gas of GT, combined cycles need to be adopted and the total investment is quite high. In the proposed hybrid scheme, HRSG and ST of WHRS for the silicon arc furnace can be used for waste heat recovery from the exhaust gas of GT, so that the equipment for the steam cycle becomes unnecessary in the plasma gasification system, saving at least 5000 k$ of investment. However, the heat exchangers in WHB need to be replaced to meet the new requirements for steam generation.

The estimated investments for adding or replacing equipment in the upgrade project are listed in Table 17. The total investment for equipment is 16191.56 k$. The plasma gasifier and the gas turbine subsystem account for most of the investment.

Table 18 shows the economic analysis results of the upgrade project of the current WHRS to the hybrid system. The upgrade costs 18,843.65 k$ for equipment purchase in the first 2 years and 1542.47 k$/year for the operation of the plasma gasifier subsystem, gas turbine system, and HXs in the following years, leading to an increase in the gross annual income of 14,189.31 k$/ye. The tipping fee for medical waste contributes to about 75% of the total income increase. Meanwhile, in the integration system, 4320 t of silica powder collected in WHB can be disposed harmlessly by the plasma gasifier every year. Although the saving from silica particle treatment is not considered, DDP is just 3.94 years since the investment, revealing good economic efficiency of this upgrade. NPV of the upgrade project in its 25-year lifetime is 61,246.12 k$. Even if the tipping fee of the waste fed into the gasifier decreases sharply to 50 $/t, DDP of the upgrade project is 16.84 years, still delivering positive returns in view of its lifetime.

The economic performance of the proposed system can be further improved by integrating two or more WHBs with one plasma gasification subsystem and one gas turbine subsystem, because the average investment for 1 MW capacity decreases as the scale of the gasifier, CC, and GT increases. The industrial base where the reference WHRS is located has 32 silicon arc furnaces and WHRS of the same design parameters, making it feasible to construct plasma gasifiers of a larger scale.

## 5. Conclusions

A novel design that integrates the current WHRS for a silicon arc furnace with plasma gasification for medical waste is proposed. In the hybrid scheme, heating of the steam and feed water in WHRS is promoted by the heat recovered from the high-temperature syngas converted from the medical waste and the exhaust gas from GT in the plasma gasification system. In view of WTE, the syngas not only drives GT for power generation but also increases power output by ST in WHRS. Meanwhile, the silica fines generated in polysilicon production can be treated harmlessly by being fed into the plasma gasifier with the medical waste. The system is modeled and simulated on EBSILON Professional and Aspen Plus platforms. Energy, exergy, and economic analyses are conducted to examine the feasibility of upgrading the current system to the proposed system.

The results indicate that in the integrated WHRS, assuming the net power output resulting from the exhaust gas leaving the arc furnace is constant, the net power generation attributed to medical waste is 4.17 MW, and the efficiency from medical waste to electricity is up to 33.99%, close to the efficiency of conventional IPGCC plants. The project of upgrading the current WHRS to the proposed hybrid system requires an initial investment of 18,843.65 k$ and attains a net annual income increase of 12,646.84 k$. DPP of the upgrade project is only 3.94 years. Meanwhile, the plasma gasifier can also dispose 4320 t of silica powder generated in the silicon arc furnace, which benefits the environmental friendliness of polysilicon production. These findings reveal good superiority and industrial prospects of the hybrid system.

## Figures and Tables

**Table 1:** Main gaseous components of the exhaust gas leaving the silicon arc furnace.

Components	N_2_	O_2_	H_2_O	CO_2_
Concentration (volume fraction)	78.48%	18.53%	1.47%	1.52%

**Table 2:** Main components of the fly ash in the exhaust gas leaving the silicon arc furnace.

Components	SiO_2_	SiC	FeO	Al_2_O_3_	CaO	MgO
Content (mass fraction)	93.30%	4.80%	0.04%	0.18%	0.40%	1.00%

**Table 3:** Assumptions used for the economic analysis of the system [22,44,60].

Item	Unit	Value
Construction period	year	2
Economic period	year	23
Annual operating time	hour	8000
Operating cost	-	10% of the total investment
Discount rate	%	12
Price of electricity	$/MWh	96.51
Price of slags	$/t	53.78
Tipping fee for medical waste	$/t	463.86

**Table 4:** Investment estimation methods for upgrading the current WHRS to the proposed hybrid system using cost function methods.

Component	Estimate Function ($)	Reference
SGC1	$C=130{\left(\frac{A}{0.093}\right)}^{0.78}$	[62]
SGC3		
SGC4		
CP1	$C=\frac{71.1m_{wf}}{0.9-\eta_{c}}r_{p}\ln(r_{p})$	[62]
CP2		
CC	$C=\frac{25.65m_{air}}{0.995-\frac{P_{out}}{P_{in}}}\left[e^{(0.018T_{out}-26.4}+1\right]$	[44]
EVA1	$C=1010{\left(A\right)}^{0.78}$	[62]
EVA2		
EG1	$C=60E_{P}{}^{0.95}$	[63]

**Table 5:** Investment estimation methods for upgrading the current WHRS to the proposed hybrid system using scaling-up methods.

Component	Basic Cost (k$)	Basic Scale	Scaling Factor	Scale Unit	Reference
Plasma gasifier	78,000.00	39.20	0.67	kg/s	[44]
Syngas cleaning section	33,650.00	4232.70	0.65	kmol/s	[64]
SGC2	45.84	500.00	0.74	m^2^	[65]
SH					
HX1					
HX2					
SGC5	44.91	500.00	0.68	m^2^	[65]
ECO					
HX3					
HX4					
HX5					
GT	1100.00	1.00	1.00	MW	[44]

**Table 6:** Properties of the medical waste fed into the plasma gasifier used for simulation (as received basis) [67].

Item		Unit	Value
Elements	C	wt%	45.71
	H	wt%	5.96
	O	wt%	37.18
	N	wt%	0.16
	S	wt%	0.12
Moisture		wt%	7.01
Ash		wt%	3.85
LHV		MJ/kg	15.35

**Table 7:** Parameters of the plasma gasifier in the proposed hybrid system.

Item		Unit	Value
Feed rate of medical waste		kg/s	0.80
Feed rate of silica powder		kg/s	0.15
Feed rate of O_2_		kg/s	0.34
Raw syngas composition	H_2_	vol%	35.35
	CO		41.18
	CH_4_		0.61
	CO_2_		12.03
	N_2_		0.08
	H_2_O		10.70
	H_2_S		0.05
Raw syngas properties	Temperature	°C	818.4
	Flow rate	kg/s	1.11
	Higher heating value	MJ/kg	11.66
	Lower heating value	MJ/kg	10.57
Output rate of slags		kg/s	0.18
Torch thermal efficiency		%	90
Torch power consumption		MW	1.43
Gasification efficiency		%	71.57

**Table 8:** Parameters of SGCs, wet scrubber, and AGR in the plasma gasifier subsystem.

Item			Unit	Value
SGC1	Syngas	Inlet temperature	°C	818.4
		Outlet temperature	°C	746.0
		Flow rate	kg/s	1.11
	Oxygen	Inlet temperature	°C	370.0
		Outlet temperature	°C	780.0
		Flow rate	kg/s	0.34
	Log mean temperature difference		°C	148.0
	Heat capacity		MW	0.15
SGC2	Syngas	Inlet temperature	°C	746.0
		Outlet temperature	°C	394.4
	Steam	Inlet temperature	°C	390.0
		Inlet pressure	MPa	3.87
		Outlet temperature	°C	480.0
		Outlet pressure	MPa	3.82
		Flow rate	kg/s	3.30
	Log mean temperature difference		°C	63.6
	Heat capacity		MW	0.69
SGC3	Syngas	Inlet temperature	°C	394.4
		Outlet temperature	°C	334.0
	Oxygen	Inlet temperature	°C	25.0
		Outlet temperature	°C	370.0
		Flow rate	kg/s	0.34
	Log mean temperature difference		°C	112.1
	Heat capacity		MW	0.11
SGC4	Syngas (cooled)	Inlet temperature	°C	334.0
		Outlet temperature	°C	133.0
	Syngas (heated)	Inlet temperature	°C	47.1
		Outlet temperature	°C	250.0
	Log mean temperature difference		°C	85.0
	Heat capacity		MW	0.37
Wet scrubber	Syngas	Inlet temperature	°C	133.0
		Outlet temperature	°C	47.1
SGC5	Syngas	Inlet temperature	°C	250.0
		Outlet temperature	°C	50.0
	Water	Inlet temperature	°C	36.2
		Outlet temperature	°C	44.8
		Flow rate	kg/s	9.91
	Log mean temperature difference		°C	71.0
	Heat capacity		MW	0.36
AGR	Syngas	Inlet temperature	°C	50.0
		Outlet temperature	°C	40.0

**Table 9:** Parameters of the gas turbine subsystem.

Item		Unit	Value
CP1 (syngas)	Inlet temperature	°C	40.0
	Inlet pressure	MPa	0.10
	Outlet temperature	°C	395.3
	Outlet pressure	MPa	1.42
	Flow rate	kg/s	1.06
	Isentropic efficiency	%	88.0
	Power consumption	MW	0.62
CP2 (O_2_)	Inlet temperature	°C	25.0
	Inlet pressure	MPa	0.10
	Outlet temperature	°C	393.9
	Outlet pressure	MPa	1.42
	Flow rate	kg/s	9.50
	Isentropic efficiency	%	88.0
	Power consumption	MW	3.67
CC	Outlet temperature	°C	1303.3
	Outlet pressure	MPa	1.41
	Flow rate	kg/s	10.56
Gas turbine	Exhaust gas temperature	°C	666.6
	Exhaust gas pressure	MPa	0.10
	Isentropic efficiency	%	90.0
	Power output	MW	4.70

**Table 10:** Main parameters of the proposed WHRS for the silicon arc furnace integrated with plasma gasification.

Item		Unit	Value
Flue gas flowing through WHB	Inlet temperature	°C	650.0
	Outlet temperature	°C	187.3
	Flow rate	kg/s	48.29
Feed water into WHB	Temperature	°C	167.0
	Pressure	MPa	4.02
	Flow rate	kg/s	9.95
Superheated steam into ST	Temperature	°C	480.0
	Pressure	MPa	3.82
	Flow rate	kg/s	9.95
Exhaust steam out of ST	Temperature	°C	36.2
	Pressure	MPa	0.01
	Flow rate	kg/s	9.90
Energy recovered from flue gas		MW	24.36
Power output by ST		MW	10.21

**Table 11:** Parameters of the heat exchangers in WHB in the proposed system.

Item			Unit	Value
Flow rate of exhaust gas from the silicon arc furnace			kg/s	48.29
ECO	Flue gas	Inlet temperature	°C	258.3
		Outlet temperature	°C	187.3
	Water	Inlet temperature	°C	167.0
		Inlet pressure	MPa	4.02
		Outlet temperature	°C	246.2
		Outlet pressure	MPa	3.92
		Flow rate	kg/s	9.95
	Log mean temperature difference		°C	15.9
	Heat capacity		MW	3.57
EVA1	Flue gas	Inlet temperature	°C	650.0
		Outlet temperature	°C	524.6
	Water/steam	Inlet temperature (water)	°C	246.2
		Inlet pressure	MPa	3.92
		Outlet temperature (steam)	°C	249.2
		Outlet pressure	MPa	3.92
		Flow rate	kg/s	3.95
	Log mean temperature difference		°C	334.2
	Heat capacity		MW	6.85
EVA2	Flue gas	Inlet temperature	°C	458.5
		Outlet temperature	°C	258.3
	Water/steam	Inlet temperature (water)	°C	246.2
		Inlet pressure	MPa	3.92
		Outlet temperature (steam)	°C	249.2
		Outlet pressure	MPa	3.92
		Flow rate	kg/s	6.00
	Log mean temperature difference		°C	63.9
	Heat capacity		MW	10.39
SH	Flue gas	Inlet temperature	°C	524.6
		Outlet temperature	°C	458.5
	Steam	Inlet temperature	°C	249.2
		Inlet pressure	MPa	3.92
		Outlet temperature	°C	450.0
		Outlet pressure	MPa	3.87
		Flow rate	kg/s	6.65
	Log mean temperature difference		°C	130.6
	Heat capacity		MW	3.53

**Table 12:** Parameters of HXs in the proposed WHRS.

Item			Unit	Value
Flow rate of combustion gas leaving GT			kg/s	10.56
HX1	Combustion gas	Inlet temperature	°C	666.6
		Outlet temperature	°C	630.0
	Steam	Inlet temperature	°C	450.0
		Inlet pressure	MPa	3.87
		Outlet temperature	°C	480.0
		Outlet pressure	MPa	3.82
		Flow rate	kg/s	6.65
	Log mean temperature difference		°C	183.3
	Heat capacity		MW	0.46
HX2	Combustion gas	Inlet temperature	°C	630.0
		Outlet temperature	°C	525.8
	Steam	Inlet temperature	°C	249.2
		Inlet pressure	MPa	3.92
		Outlet temperature	°C	390.0
		Outlet pressure	MPa	3.87
		Flow rate	kg/s	3.30
	Log mean temperature difference		°C	257.9
	Heat capacity		MW	1.29
HX3	Combustion gas	Inlet temperature	°C	525.8
		Outlet temperature	°C	304.4
	Water	Inlet temperature	°C	104.8
		Inlet pressure	MPa	4.12
		Outlet temperature	°C	167.0
		Outlet pressure	MPa	4.02
		Flow rate	kg/s	9.95
	Log mean temperature difference		°C	271.5
	Heat capacity		MW	2.64
HX4	Combustion gas	Inlet temperature	°C	304.4
		Outlet temperature	°C	155.0
	Water	Inlet temperature	°C	61.4
		Inlet pressure	MPa	0.12
		Outlet temperature	°C	102.2
		Outlet pressure	MPa	0.12
		Flow rate	kg/s	9.91
	Log mean temperature difference		°C	141.0
	Heat capacity		MW	1.70
HX5	Combustion gas	Inlet temperature	°C	155.0
		Outlet temperature	°C	95.0
	Water	Inlet temperature	°C	44.8
		Inlet pressure	MPa	0.13
		Outlet temperature	°C	60.9
		Outlet pressure	MPa	0.13
		Flow rate	kg/s	9.91
	Log mean temperature difference		°C	69.9
	Heat capacity		MW	0.67

**Table 13:** Parameters of the equipment in the heat regeneration subsystem.

Item			Unit	Value
COND	Inlet steam	Temperature	°C	36.2
		Pressure	MPa	0.01
		Flow rate	kg/s	9.90
	Outlet condensed water	Temperature	°C	36.2
		Pressure	MPa	0.01
		Flow rate	kg/s	9.91
RH	Inlet feed water	Temperature	°C	60.9
		Pressure	MPa	0.13
		Flow rate	kg/s	9.91
	Extracted steam from ST	Temperature	°C	67.5
		Pressure	MPa	0.03
		Flow rate	kg/s	0.01
	Outlet feed water	Temperature	°C	61.4
		Pressure	MPa	0.12
		Flow rate	kg/s	9.91
	Drain water	Temperature	°C	36.2
		Pressure	MPa	0.01
		Flow rate	kg/s	0.01
DEA	Inlet feed water	Temperature	°C	102.2
		Pressure	MPa	0.12
		Flow rate	kg/s	9.91
	Extracted steam from ST	Temperature	°C	133.1
		Pressure	MPa	0.14
		Flow rate	kg/s	0.04
	Outlet feed water	Temperature	°C	104.3
		Pressure	MPa	4.12
		Flow rate	kg/s	9.95

**Table 14:** Energy performance of the proposed integrated system, compared with the current WHRS.

Item	Unit	Proposed Integrated System	Current WHRS
Energy input by exhaust gas from the silicon arc furnace	MW	33.59	33.59
Energy input by the medical waste	MW	12.28	/
Power output by ST	MW	10.21	8.08
Power output by GT	MW	4.70	/
Power consumption by auxiliaries in WHRS	MW	1.21	1.21
Power consumption by torches	MW	1.43	/
Power consumption by syngas conditioning equipment	MW	0.23	/
Power consumption by O_2_ separation	MW	0.32	
Power consumption by CP1	MW	0.62	/
Total auxiliary power consumption	MW	3.82	1.21
Total net power output	MW	11.04	6.87
Net power generation by exhaust gas from the silicon arc furnace	MW	6.87	6.87
Net power generation by medical waste	MW	4.17	/
Net power generation efficiency by medical waste	%	33.99	/
Overall power generation efficiency	%	30.13	28.19

**Table 15:** Exergy performance of the proposed WHRS, compared with the current WHRS.

Item	Unit	Proposed Integrated System	Current WHRS
Exergy input by the flue gas from the silicon arc furnace	MW	15.07	15.07
	%	100.00	100.00
Exergy input by medical waste	MW	13.22	/
	%	87.75	/
Total exergy input	MW	28.29	15.07
	%	187.75	100.00
Exergy output by exhaust gas from silicon arc furnace (electricity)	MW	6.87	6.87
	%	45.58	45.58
Exergy output by medical waste (electricity)	MW	4.17	/
	%	27.71	/
Total exergy output (electricity)	MW	11.03	6.87
	%	73.29	45.58
Exergy efficiency of power generation by medical waste	%	31.58	/
Overall exergy efficiency	%	39.04	45.58

**Table 16:** Exergy destruction in the proposed system, compared with the current WHRS.

Item		Unit	Proposed Integrated System	Current WHRS
Plasma gasifier subsystem	Gasifier	MW	2.559	/
	SGC1	MW	0.011	/
	SGC2	MW	0.047	/
	SGC3	MW	0.022	/
	SGC4	MW	0.046	/
	Wet scrubber	MW	0.050	/
	SGC5	MW	0.060	
	AGR	MW	0.038	/
Gas turbine system	CP1	MW	0.040	/
	CP2	MW	0.238	/
	CC	MW	2.674	/
	GT	MW	0.395	/
	EG1	MW	0.047	/
WHB		MW	4.517	4.78
ST		MW	1.835	1.45
EG2		MW	0.103	0.08
Heat regeneration subsystem	COND	MW	0.789	0.59
	RH	MW	0.001	0.04
	DEA	MW	0.001	0.05
HXs	HX1	MW	0.049	/
	HX2	MW	0.212	/
	HX3	MW	0.733	/
	HX4	MW	0.416	/
	HX5	MW	0.596	/
Auxiliaries in WHRS		MW	1.764	1.21
Sum		MW	17.25	8.20

**Table 17:** Investment estimation of equipment for upgrading the current WHRS to the proposed hybrid system.

Item		Cost(k$)
Plasma gasifier subsystem	Gasifier	6451.60
	SGC1	17.53
	SGC2	36.05
	SGC3	17.66
	SGC4	54.71
	SGC5	17.45
	Syngas cleaning section	22.85
Gas turbine system	CP1	139.37
	CP2	1247.79
	CC	1948.52
	GT	5169.35
	EG1	183.00
WHB	EVA1	76.39
	EVA2	384.33
	SH	71.00
	ECO	235.15
HXs	HX1	12.22
	HX2	20.34
	HX3	27.43
	HX4	31.77
	HX5	27.05
Total investment of equipment for upgrade		16,191.56

**Table 18:** Economic performance of upgrading the current WHRS to the hybrid system.

Item	Unit	Value
Investment for equipment	k$	16,191.56
Compensation for income loss of current WHRS	k$	2652.09
Annual operating cost increase	k$	1542.47
Medical waste treatment capacity	t/year	23,040.00
Income from medical waste treatment	k$/year	10,687.33
Power generation increase	MWh/year	33,397.38
Income increase from electricity selling	k$/year	3223.18
Slag production	t/year	5184.00
Income from slag selling	k$/year	278.80
Gross annual income increase	k$/year	14,189.31
Net annual income increase	k$/year	12,646.84
Dynamic payback period (DDP)	year	3.94
Net present value (NPV)	k$	61,246.12

## Data Availability

Not applicable.

## Nomenclature

*C*	Cash
*EX*	Exergy
*P*	Power
*Q*	Thermal energy
*T*	Temperature
*W*	Work
*h*	Specific enthalpy
*m*	Mass flow
*r*	Rate
*ω*	Content
*η*	Efficiency
**Superscripts and Subscripts**	
0	environmental state
dis	Discount
des	Destruction
eg	Energy
ex	Exergy
fg	Flue gas
gr	gross
in	inlet
mw	Medical waste
out	outlet
p	Power
pg	Plasma gasification
rec	Recovery
syn	Syngas
tor	Torch
tot	Total
wh	Waste heat recovery system
**Abbreviations**	
AGR	Acid gas removal
CC	Combustion chamber
COND	Condenser
COS	Carbonyl sulfide
CP	Compressor
CS	Cyclone separator
DC	Direct current
DDP	Dynamic payback period
DEA	Deaerator
ECO	Economizer
EG	Electric generator
EVA	Evaporator
GT	Gas turbine
HRSG	Heat recovery steam generator
HX	Heat exchanger
IPGCC	Integrated plasma gasification combined cycle
LHV	Lower calorific value
MDEA	Methyl diethanolamine
MSW	Municipal solid wastes
MW	Medical waste
NPV	Net present value
PV	Photovoltaics
RH	Regenerative heater
SGC	Syngas cooler
SH	Superheater
ST	Steam turbine
WHB	Waste heat boiler
WHRS	Waste heat recovery system
WTE	Waste to energy

## Appendix A

**Table A1:** Simulation results of the current WHRS for the silicon arc furnace modeled by EBSILON Professional, compared with actual parameters.

Item		Unit	Reference Value	Simulation Value
Flue gas through WHB	Inlet temperature	°C	650.0	650.0
	Outlet temperature	°C	185.0	187.3
	Flow rate	kg/s	48.29	48.29
Feed water into WHB	Temperature	°C	104.0	104.8
	Pressure	MPa	4.02	4.02
	Flow rate	kg/s	8.33	8.33
Superheated steam into ST	Temperature	°C	450.0	450.0
	Pressure	MPa	3.82	3.82
	Flow rate	kg/s	8.43	8.43
Exhaust steam of ST	Temperature	°C	36.2	36.2
	Pressure	MPa	0.01	0.01
	Flow rate	kg/s	7.43	7.43
Energy recovered from flue gas		MW	24.46	24.36
Gross power output		MW	8.12	8.08
Auxiliary power consumption		MW	1.21	1.21
Net power output		MW	6.91	6.87
Power generation efficiency		%	28.35	28.19

**Table A2:** Properties of the main streams in the current WHRS in Figure 1.

Stream No.	Substance	Temperature (°C)	Pressure (MPa)	Flow Rate (kg/s)
1	Flue gas	650.0	0.10	48.29
2	Flue gas	597.0	0.10	48.29
3	Flue gas	514.4	0.10	48.29
4	Flue gas	291.5	0.10	48.29
5	Flue gas	187.3	0.10	48.29
6	Water	104.8	4.02	8.43
7	Water	246.2	3.92	8.43
8	Water	246.2	3.92	8.43
9	Water	246.2	3.92	6.75
10	Water	246.2	3.92	1.68
11	Steam	249.2	3.92	6.75
12	Steam	249.2	3.92	1.68
13	Steam	249.2	3.92	8.43
14	Steam	450.0	3.82	8.43
15	Steam	113.4	0.14	0.62
16	Steam	67.5	0.03	0.37
17	Steam	36.2	0.01	7.43
18	Water	36.2	0.12	7.81
19	Water	36.2	0.01	0.37
20	Water	61.4	0.12	7.81

**Table A3:** Simulation results of a plasma gasifier fueled with solid waste modeled by Aspen Plus, compared with reference parameters [68].

Item		Unit	Reference Value	Simulation Value
Feed rate of waste		kg/s	1.00	1.00
Lower heating value of waste		MJ/kg	23.67	23.67
Feed rate of air		kg/s	0.16	0.16
Feed rate of steam		kg/s	0.20	0.20
Raw syngas composition	H_2_	vol%	43.50	43.16
	CO		34.50	33.37
	CH_4_		0.01	0.56
	CO_2_		0.03	0.90
	N_2_		5.63	5.73
	H_2_O		16.22	16.18
	H_2_S		0.09	0.08
Raw syngas properties	Temperature	°C	1267.0	1204.1
	Flow rate	kg/s	1.21	1.21
	Higher heating value	MJ/kg	14.71	14.64
	Lower heating value	MJ/kg	13.44	13.36
Slag output		kg/s	0.15	0.15
Torch power consumption		MW	4.06	4.08
Torch thermal efficiency		%	86.0	86.0
Plasma gasification efficiency		%	43.30	44.11

**Table A4:** Simulation results of CPs, CC, and GT modeled by EBSILON Professional, compared with reference parameters [69].

Item		Unit	Reference Value	Simulation Value
CP (air)	Inlet temperature	°C	25.0	25.0
	Inlet pressure	MPa	0.10	0.10
	Compression ratio	-	14.87	14.87
	Flow rate	kg/s	73.23	73.23
	Isentropic efficiency [70]	%	88.0	88.0
	Power consumption	MW	29.12	29.34
CC	Outlet temperature	°C	1247.0	1242.2
	Outlet pressure	MPa	14.27	14.27
	Flow rate	kg/s	74.90	74.83
Gas turbine	Exhaust gas temperature	°C	631.0	627.9
	Exhaust gas pressure	MPa	0.11	0.11
	Exhaust gas flow rate	kg/s	74.90	74.83
	Isentropic efficiency	%	90.0	90.0
	Power output	MW	58.61	58.29

**Table A5:** Properties of the main streams in the hybrid scheme in Figure 2.

Stream No.	Substance	Temperature (°C)	Pressure (MPa)	Flow Rate (kg/s)
1	Flue gas	650.0	0.10	48.29
2	Flue gas	524.6	0.10	48.29
3	Flue gas	458.5	0.10	48.29
4	Flue gas	258.3	0.10	48.29
5	Flue gas	187.3	0.10	48.29
6	Oxygen	25.0	0.10	0.34
7	Oxygen	370.0	0.10	0.34
8	Oxygen	780.0	0.10	0.34
9	Syngas	818.4	0.10	1.11
10	Syngas	746.0	0.10	1.11
11	Syngas	394.4	0.10	1.11
12	Syngas	334.0	0.10	1.11
13	Syngas	133.0	0.10	1.11
14	Syngas	47.1	0.10	1.11
15	Syngas	250.0	0.10	1.11
16	Syngas	50.0	0.10	1.11
17	Syngas	40.0	0.10	1.06
18	Air	25.0	0.10	9.50
19	Air	393.9	1.42	9.50
20	Syngas	395.3	1.42	1.06
21	Combustion gas	1303.3	1.41	10.56
22	Combustion gas	666.6	0.10	10.56
23	Combustion gas	630.0	0.10	10.56
24	Combustion gas	525.8	0.10	10.56
25	Combustion gas	304.4	0.10	10.56
26	Combustion gas	155.0	0.10	10.56
27	Combustion gas	95.0	0.10	10.56
28	Water	104.8	4.12	9.95
29	Water	167.0	4.02	9.95
30	Water	246.2	3.92	9.95
31	Water	249.2	3.92	9.95
32	Water	249.2	3.92	3.95
33	Water	249.2	3.92	6.00
34	Steam	249.2	3.92	3.95
35	Steam	249.2	3.92	6.00
36	Steam	249.2	3.92	9.95
37	Steam	249.2	3.92	3.30
38	Steam	249.2	3.92	6.65
39	Steam	390.0	3.87	3.30
40	Steam	450.0	3.87	6.65
41	Steam	480.0	3.82	3.30
42	Steam	480.0	3.82	6.65
43	Steam	480.0	3.82	9.95
44	Steam	133.1	0.14	0.04
45	Steam	67.5	0.03	0.01
46	Steam	36.2	0.01	9.90
47	Water	36.2	0.14	9.91
48	Water	44.8	0.13	9.91
49	Water	60.9	0.13	9.91
50	Water	36.2	0.01	0.01
51	Water	61.4	0.12	9.91
52	Water	102.2	0.12	9.91

## References

[1] IEA (2021). Renewables.

[2] Shen Z., Ma L., Yang Y., Fan M., Ma W., Fu L., Li M. (2022). A life cycle assessment of hydropower-silicon-photovoltaic industrial chain in china. J. Clean Prod..

[3] Xin-Gang Z., You Z. (2018). Technological progress and industrial performance: A case study of solar photovoltaic industry. Renew. Sustain. Energy Rev..

[4] IEA (2022). Solar PV Global Supply Chains.

[5] Eriksson G., Hack K., Hack K. (2008). Iv.7—Production of metallurgical-grade silicon in an electric arc furnace. The SGTE Casebook.

[6] Satpathy R., Pamuru V., Satpathy R., Pamuru V. (2021). Chapter 1—Manufacturing of polysilicon. Solar PV Power.

[7] Chigondo F. (2018). From metallurgical-grade to solar-grade silicon: An overview. Silicon.

[8] Chen Z., Ma W., Wu J., Wei K., Lei Y., Lv G. (2018). A study of the performance of submerged arc furnace smelting of industrial silicon. Silicon.

[9] Takla M., Kamfjord N.E., Tveit H., Kjelstrup S. (2013). Energy and exergy analysis of the silicon production process. Energy.

[10] Filtvedt W.O., Javidi M., Holt A., Melaaen M.C., Marstein E., Tathgar H., Ramachandran P.A. (2010). Development of fluidized bed reactors for silicon production. Sol. Energy Mater. Sol. Cells.

[11] Hong J., Chen W., Qi C., Ye L., Xu C. (2016). Life cycle assessment of multicrystalline silicon photovoltaic cell production in china. Sol. Energy.

[12] Liu F., van den Bergh J.C.J.M. (2020). Differences in CO_2_ emissions of solar PV production among technologies and regions: Application to China, EU and USA. Energy Policy.

[13] Zhang P., Duan J., Chen G., Li J., Wang W. (2018). Production of polycrystalline silicon from silane pyrolysis: A review of fines formation. Sol. Energy.

[14] Nemchinova N.V., Mineev G.G., Tyutrin A.A., Yakovleva A.A. (2017). Utilization of dust from silicon production. Steel Transl..

[15] Tavares R., Ramos A., Rouboa A. (2019). A theoretical study on municipal solid waste plasma gasification. Waste Manag..

[16] Li J., Liu K., Yan S., Li Y., Han D. (2016). Application of thermal plasma technology for the treatment of solid wastes in China: An overview. Waste Manag..

[17] Gomez E., Rani D.A., Cheeseman C.R., Deegan D., Wise M., Boccaccini A.R. (2009). Thermal plasma technology for the treatment of wastes: A critical review. J. Hazard. Mater..

[18] Ramos A., Rouboa A. (2022). Life cycle thinking of plasma gasification as a waste-to-energy tool: Review on environmental, economic and social aspects. Renew. Sustain. Energy Rev..

[19] Zhang Q., Wu Y., Dor L., Yang W., Blasiak W. (2013). A thermodynamic analysis of solid waste gasification in the plasma gasification melting process. Appl. Energy.

[20] Montiel-Bohórquez N.D., Saldarriaga-Loaiza J.D., Pérez J.F. (2022). Analysis of investment incentives for power generation based on an integrated plasma gasification combined cycle power plant using municipal solid waste. Case Stud. Therm. Eng..

[21] Mountouris A., Voutsas E., Tassios D. (2006). Solid waste plasma gasification: Equilibrium model development and exergy analysis. Energy Conv. Manag..

[22] Danthurebandara M., Van Passel S., Machiels L., Van Acker K. (2015). Valorization of thermal treatment residues in enhanced landfill mining: Environmental and economic evaluation. J. Clean Prod..

[23] Sanito R.C., Bernuy-Zumaeta M., You S., Wang Y. (2022). A review on vitrification technologies of hazardous waste. J. Environ. Manag..

[24] Minutillo M., Perna A., Di Bona D. (2009). Modelling and performance analysis of an integrated plasma gasification combined cycle (IPGCC) power plant. Energy Conv. Manag..

[25] Fabry F., Rehmet C., Rohani V., Fulcheri L. (2013). Waste gasification by thermal plasma: A review. Waste Biomass Valoriz..

[26] Munir M.T., Mardon I., Al-Zuhair S., Shawabkeh A., Saqib N.U. (2019). Plasma gasification of municipal solid waste for waste-to-value processing. Renew. Sustain. Energy Rev..

[27] Subramanyam V., Gorodetsky A., Wang T., Stiegel G. (2017). 5–municipal wastes and other potential fuels for use in igcc systems. Integrated Gasification Combined Cycle (IGCC) Technologies.

[28] Awasthi S.K., Sarsaiya S., Kumar V., Chaturvedi P., Sindhu R., Binod P., Zhang Z., Pandey A., Awasthi M.K. (2022). Processing of municipal solid waste resources for a circular economy in China: An overview. Fuel.

[29] Valmundsson A.S., Janajreh I. Plasma gasification process modeling and energy recovery from solid waste. Proceedings of the ASME 5th International Conference on Energy Sustainability.

[30] Cudjoe D., Wang H. (2022). Plasma gasification versus incineration of plastic waste: Energy, economic and environmental analysis. Fuel Process. Technol..

[31] James I.R.E., Keairns D., Turner M., Woods M., Kuehn N., Zoelle A. (2019). Cost and Performance Baseline for Fossil Energy Plants Volume 1: Bituminous Coal and Natural Gas to Electricity.

[32] Robil Zwart S.V.D.H. (2010). Tar Removal from Low-Temperature Gasifiers.

[33] Pérez-Fortes M., Bojarski A.D., Velo E., Nougués J.M., Puigjaner L. (2009). Conceptual model and evaluation of generated power and emissions in an IGCC plant. Energy.

[34] Montiel-Bohórquez N.D., Agudelo A.F., Pérez J.F. (2021). Effect of origin and production rate of msw on the exergoeconomic performance of an integrated plasma gasification combined cycle power plant. Energy Conv. Manag..

[35] Mazzoni L., Janajreh I. (2017). Plasma gasification of municipal solid waste with variable content of plastic solid waste for enhanced energy recovery. Int. J. Hydrogen Energy.

[36] Erdogan A.A., Yilmazoglu M.Z. (2021). Plasma gasification of the medical waste. Int. J. Hydrogen Energy.

[37] Sikarwar V.S., Hrabovský M., Van Oost G., Pohořelý M., Jeremiáš M. (2020). Progress in waste utilization via thermal plasma. Prog. Energy Combust. Sci..

[38] Su G., Ong H.C., Ibrahim S., Fattah I.M.R., Mofijur M., Chong C.T. (2021). Valorisation of medical waste through pyrolysis for a cleaner environment: Progress and challenges. Environ. Pollut..

[39] Deng N., Zhang Y., Wang Y. (2008). Thermogravimetric analysis and kinetic study on pyrolysis of representative medical waste composition. Waste Manag..

[40] Du Y., Ju T., Meng Y., Lan T., Han S., Jiang J. (2021). A review on municipal solid waste pyrolysis of different composition for gas production. Fuel Process. Technol..

[41] Ding Z., Chen H., Liu J., Cai H., Evrendilek F., Buyukada M. (2021). Pyrolysis dynamics of two medical plastic wastes: Drivers, behaviors, evolved gases, reaction mechanisms, and pathways. J. Hazard. Mater..

[42] Saxena P., Pradhan I.P., Kumar D. (2022). Redefining bio medical waste management during COVID-19 in india: A way forward. Mat. Today Proc..

[43] Windfeld E.S., Brooks M.S. (2015). Medical waste management—A review. J. Environ. Manag..

[44] Chen H., Li J., Li T., Xu G., Jin X., Wang M., Liu T. (2022). Performance assessment of a novel medical-waste-to-energy design based on plasma gasification and integrated with a municipal solid waste incineration plant. Energy.

[45] Yang Y., Wang J., Chong K., Bridgwater A.V. (2018). A techno-economic analysis of energy recovery from organic fraction of municipal solid waste (MSW) by an integrated intermediate pyrolysis and combined heat and power (CHP) plant. Energy Conv. Manag..

[46] Pan P., Peng W., Li J., Chen H., Xu G., Liu T. (2022). Design and evaluation of a conceptual waste-to-energy approach integrating plasma waste gasification with coal-fired power generation. Energy.

[47] Courson C., Gallucci K., Materazzi M., Foscolo P.U. (2019). 8—Gas cleaning for waste applications (syngas cleaning for catalytic synthetic natural gas synthesis). Substitute Natural Gas from Waste.

[48] Woolcock P.J., Brown R.C. (2013). A review of cleaning technologies for biomass-derived syngas. Biomass Bioenerg..

[49] Kosstrin H.M., Wang T., Stiegel G. (2017). 10–wet scrubbing and gas filtration of syngas in igcc systems. Integrated Gasification Combined Cycle (IGCC) Technologies.

[50] Sharma S.D., Dolan M., Park D., Morpeth L., Ilyushechkin A., Mclennan K., Harris D.J., Thambimuthu K.V. (2008). A critical review of syngas cleaning technologies—Fundamental limitations and practical problems. Powder Technol..

[51] Giuffrida A., Romano M.C. On the effects of syngas clean-up temperature in IGCCs. Proceedings of the ASME Turbo Exp 2010.

[52] Frilund C., Tuomi S., Kurkela E., Simell P. (2021). Small- to medium-scale deep syngas purification: Biomass-to-liquids multi-contaminant removal demonstration. Biomass Bioenerg..

[53] Huang H., Young N., Williams B.P., Taylor S.H., Hutchings G. (2006). High temperature cos hydrolysis catalysed by gamma-Al_2_O_3_. Catal. Lett..

[54] Shangguan J., Liu Y., Wang Z., Xu Y. (2019). The synthesis of magnesium-aluminium spinel for catalytic hydrolysis of carbonyl sulphur at the middle temperature. IOP Conf. Ser. Mat. Sci. Eng..

[55] Al Ani Z., Thafseer M., Gujarathi A.M., Vakili-Nezhaad G.R. (2020). Towards process, energy and safety based criteria for multi-objective optimization of industrial acid gas removal process. Process Saf. Environ. Protect..

[56] Bhattacharyya D., Turton R., Zitney S.E., Wang T., Stiegel G. (2017). 11–acid gas removal from syngas in IGCC plants. Integrated Gasification Combined Cycle (IGCC) Technologies.

[57] Indrawan N., Mohammad S., Kumar A., Huhnke R.L. (2019). Modeling low temperature plasma gasification of municipal solid waste. Environ. Technol. Innov..

[58] Chaiyat N. (2021). Energy, exergy, economic, and environmental analysis of an organic rankine cycle integrating with infectious medical waste incinerator. Therm. Sci. Eng. Prog..

[59] Mahian O., Mirzaie M.R., Kasaeian A., Mousavi S.H. (2020). Exergy analysis in combined heat and power systems: A review. Energy Conv. Manag..

[60] Danthurebandara M., Van Passel S., Vanderreydt I., Van Acker K. (2015). Environmental and economic performance of plasma gasification in enhanced landfill mining. Waste Manag..

[61] Zang G., Jia J., Tejasvi S., Ratner A., Lora E.S. (2018). Techno-economic comparative analysis of Biomass Integrated Gasification Combined Cycles with and without CO_2_ capture. Int. J. Greenh. Gas Control.

[62] Ogorure O.J., Oko C.O.C., Diemuodeke E.O., Owebor K. (2018). Energy, exergy, environmental and economic analysis of an agricultural waste-to-energy integrated multigeneration thermal power plant. Energy Conv. Manag..

[63] Lian Z.T., Chua K.J., Chou S.K. (2010). A thermoeconomic analysis of biomass energy for trigeneration. Appl. Energy.

[64] Peng W., Chen H., Liu J., Zhao X., Xu G. (2021). Techno-economic assessment of a conceptual waste-to-energy CHP system combining plasma gasification, SOFC, gas turbine and supercritical CO_2_ cycle. Energy Conv. Manag..

[65] Elsido C., Martelli E., Kreutz T. (2019). Heat integration and heat recovery steam cycle optimization for a low-carbon lignite/biomass-to-jet fuel demonstration project. Appl. Energy.

[66] Zhao X., Jiang G., Li A., Wang L. (2016). Economic analysis of waste-to-energy industry in China. Waste Manag..

[67] Zhu H.M., Yan J.H., Jiang X.G., Lai Y.E., Cen K.F. (2008). Study on pyrolysis of typical medical waste materials by using TG-FTIR analysis. J. Hazard. Mater..

[68] Janajreh I., Raza S.S., Valmundsson A.S. (2013). Plasma gasification process: Modeling, simulation and comparison with conventional air gasification. Energy Conv. Manag..

[69] Hou S., Zhou Y., Yu L., Zhang F., Cao S., Wu Y. (2018). Optimization of a novel cogeneration system including a gas turbine, a supercritical CO_2_ recompression cycle, a steam power cycle and an organic Rankine cycle. Energy Conv. Manag..

[70] Roy D., Samanta S., Ghosh S. (2020). Performance assessment of a biomass-fuelled distributed hybrid energy system integrating molten carbonate fuel cell, externally fired gas turbine and supercritical carbon dioxide cycle. Energy Conv. Manag..

